# Italian association of clinical endocrinologists (AME) position statement: drug therapy of osteoporosis

**DOI:** 10.1007/s40618-016-0434-8

**Published:** 2016-03-11

**Authors:** F. Vescini, R. Attanasio, A. Balestrieri, F. Bandeira, S. Bonadonna, V. Camozzi, S. Cassibba, R. Cesareo, I. Chiodini, C. Maria Francucci, L. Gianotti, F. Grimaldi, R. Guglielmi, B. Madeo, C. Marcocci, A. Palermo, A. Scillitani, E. Vignali, V. Rochira, M. Zini

**Affiliations:** 1 Endocrinology and Metabolic Disease Unit, Azienda Ospedaliero-Universitaria Santa Maria della Misericordia, P.le S.M. della Misericordia, 15, 33100 Udine, Italy; 2 Endocrinology Service, Galeazzi Institute IRCCS, Milan, Italy; 3 Unit of Endocrinology and Diabetology, Department of Internal Medicine, M. Bufalini Hospital, Cesena, Italy; 4 Division of Endocrinology, Diabetes and Bone Diseases, Agamenon Magalhães Hospital, University of Pernambuco Medical School, Recife, Brazil; 5 Istituto Auxologico Italiano, Milan, Italy; 6 Unit of Endocrinology, Department of Medicine, University of Padova, Padua, Italy; 7 Endocrinology and Diabetology, Papa Giovanni XXIII Hospital, Bergamo, Italy; 8 Endocrinology, S. Maria Goretti Hospital, Latina, Italy; 9 Department of Clinical Sciences and Community Health, University of Milan, Milan, Italy; 10 Post Acute and Long Term Care Department, I.N.R.C.A., Ancona, Italy; 11 San Pier Damiano Hospital, Villa Maria Group Care and Research, Faenza, Ravenna Italy; 12 Endocrinology and Metabolic Diseases, S. Croce e Carle Hospital, Cuneo, Italy; 13 Endocrinology Unit, Regina Apostolorum Hospital, Albano Laziale, Rome Italy; 14 Integrated Department of Medicine, Endocrinology and Metabolism, Geriatrics, University of Modena and Reggio Emilia, Modena, Italy; 15 Endocrine Unit 2, Department of Clinical and Experimental Medicine, University Hospital of Pisa, Pisa, Italy; 16 Department of Endocrinology and Diabetes, University Campus Bio-Medico, Rome, Italy; 17 Endocrinology, Casa Sollievo della Sofferenza IRCCS, San Giovanni Rotondo, Italy; 18 Department of Clinical and Experimental Medicine, University Hospital of Pisa, Pisa, Italy; 19 Unit of Endocrinology, Department of Biomedical, Metabolic and Neural Sciences, University of Modena and Reggio Emilia, Modena, Italy; 20 Endocrinology Unit, Arcispedale S. Maria Nuova IRCCS, Reggio Emilia, Italy

**Keywords:** Osteoporosis, Fracture, Treatment, Bisphosphonates, Teriparatide, Strontium ranelate, Denosumab, SERMs, Adherence, Side effects, Non-responder, Male osteoporosis, Drug-induced osteoporosis, Androgen deprivation, Aromatase inhibitors, Length of therapy

## Abstract

Treatment of osteoporosis is aimed to prevent fragility fractures and to stabilize or increase bone mineral density. Several drugs with different efficacy and safety profiles are available. The long-term therapeutic strategy should be planned, and the initial treatment should be selected according to the individual site-specific fracture risk and the need to give the maximal protection when the fracture risk is highest (i.e. in the late life). The present consensus focused on the strategies for the treatment of postmenopausal osteoporosis taking into consideration all the drugs available for this purpose. A short revision of the literature about treatment of secondary osteoporosis due both to androgen deprivation therapy for prostate cancer and to aromatase inhibitors for breast cancer was also performed. Also premenopausal females and males with osteoporosis are frequently seen in endocrine settings. Finally particular attention was paid to the tailoring of treatment as well as to its duration.

**Electronic supplementary material:**

The online version of this article (doi:10.1007/s40618-016-0434-8) contains supplementary material, which is available to authorized users.

## Table of contents

**Table:** 

Abstract	Monitoring the effectiveness of treatment
Introduction	Bone turnover markers
Methodology	Bone mineral density
Postmenopausal osteoporosis	Conducting the treatment
Whom to treat	Defining a non-responder
Risk factors	How long to treat
Treatment thresholds and indications to treatment	Drug switch
How to treat	Adherence
Available drugs	Other forms of osteoporosis
Bisphosphonates	Male osteoporosis
Alendronate	Androgen deprivation therapy
Risedronate	Premenopausal osteoporosis
Ibandronate	Aromatase inhibitors
Zoledronate	Conclusions
Clodronate	References
Strontium ranelate	
Denosumab	
SERMs	
Hormone therapy	
Teriparatide	

## Introduction

The goals of treatment of osteoporosis are to prevent fragility fractures and to stabilize or increase bone mineral density (BMD). Several drugs with different efficacy and safety profiles are now available, but no drug can be indefinitely used once started. The long-term therapeutic strategy should be planned, and the initial treatment selected according to the individual site-specific fracture risk when therapy is started and the need to give the maximal protection when the fracture risk is highest (i.e. later in life).

Regardless of the drug used, adequate calcium intake and vitamin D status are prerequisites of any osteoporosis prevention and treatment program [1].

The aim of the present consensus was mainly to review the available literature on the drug treatment of postmenopausal osteoporosis (PMO); some particular forms of secondary osteoporosis were reviewed as well. Our purpose was to provide endocrinologists with an easy tool for the management of osteoporotic patients in their clinical practice, thus we did not address epidemiology, classification, pathogenesis.

## Methodology

The methodology of the present Consensus is based upon the Grading of Recommendations, Assessment, Development, and Evaluation (GRADE) system [2–4].

According to the GRADE system, the evidence quality is categorized as *high, moderate, low, or very low.* High quality evidence is defined as consistent evidence from well-performed randomized controlled trials (RCT) or exceptionally strong evidence from unbiased observational studies. Moderate quality evidence is evidence from RCTs with important limitations (inconsistent results, methodological flaws, indirect or imprecise evidence), or unusually strong evidence from unbiased observational studies. Low-quality evidence derives from observational studies, from RCTs with serious flaws, or indirect evidence. Very low-quality evidence stems from unsystematic clinical observations or very indirect evidence.

The GRADE system classifies the strength of recommendations into two grades (*strong* or *weak*). Strong recommendations (terminology: “*we recommend*”) mean that benefits clearly outweigh harms and burdens, or vice versa. Weak recommendations (terminology: “*we suggest*”) mean that the desirable effects of adherence to a recommendation probably outweigh the undesirable effects, but the panel is not confident.

Basically, high-level evidence supports strong recommendations, whereas biased or low-quality evidences generate weak recommendations. However, making recommendations for practice purposes require taking into account other factors, such as patients’ values and preferences, local circumstances, and clinical expertise. As a consequence, the strength of a recommendation can be downgraded (weak recommendation generated by high or moderate quality evidence) or upgraded (strong recommendation generated from low or very low-quality evidence). The GRADE system formally recognizes this possibility. For example, poor quality of planning of a study suggesting high likelihood of bias, inconsistency of results, indirectness of evidence, surrogate or weak end-points, lack of precision with large confidence intervals may induce the panel to reduce the strength of a recommendation based upon an RCT. Vice versa, a recommendation based upon observational studies can be upgraded in case of large magnitude of effect, good quality of the study, long follow-up, large cohorts of patients.

## Postmenopausal osteoporosis (PMO)

### Whom to treat

#### Risk factors

To prevent the fracture, any fracture risk should be evaluated in the individual patient, adjusting all the modifiable ones [5, 6] (table I in supplemental material).

*BMD* is the major risk factor especially in over 65-year women. A decrease in BMD of about 1 SD represents an increased risk factor ranging from 1.5 to 2.5, depending mostly on the capacity of the BMD to predict the fracture risk on the same site. This relationship decreases with age, especially for hip fracture [7].

*Puberty* is a crucial period characterized by bone mass acquisition. Any adverse event affecting puberty directly (e.g. delayed puberty, hypogonadism) or indirectly (e.g. anorexia nervosa, starvation, excess physical activity) might permanently compromise peak bone mass acquisition both quantitative and qualitative [7–9].

*Aging* makes risk fracture increase, especially at the hip. Over 50 years, postmenopausal women double the risk of fractures every 7 or 8 years, being 70 and 82 years the median age for vertebral fracture and hip fracture, respectively [10]. In men, the risk of fractures increases later, becoming clinically relevant after 65–70 years [11, 12].

Genetic factors seem to be the principal determinants in peak bone mass. Patients whose first-degree relatives are osteoporotic or suffered prior fracture have a lower BMD and increased fracture risk [13].

A *prior osteoporotic fracture* induces an increased risk for future fractures: prior forearm fracture is associated with a twofold increased risk of another fracture [14] and the risk of future vertebral deformities over 3 years is fivefold higher in patients with prior deformities. The risk of recurrent fracture is higher as the number of preexisting fractures increases, regardless of BMD adjusting.

Low *body mass index* (BMI < 18 kg/m^2^) is a risk factor for low BMD, and, in older women, thinness is associated with increased fracture risk [15].

Several *lifestyle factors* negatively affect BMD and fracture risk: cigarette smoking, heavy alcohol consumption, absence of physical activity, low calcium and/or vitamin D intake, high caffeine intake [16].

The reduction of estrogens after *menopause* increases bone resorption, mostly after 3–4 years. In the first 5–7 years after menopause bone loss is estimated to be about 10 % at the spine, 5 % at the femoral neck (FN), and 7 % in the whole body. With aging, postmenopausal women also develop an increased fracture risk ratio. Women with early-onset menopause (before 40 years) are at greater risk of developing osteoporosis [17].

*Secondary causes* of fracture risk are specific medications and chronic or genetic diseases (table II in supplemental material) [5, 6].

Secondary osteoporosis occurs in almost two-thirds of men, more than half of premenopausal and perimenopausal women, and about one-fifth of postmenopausal women. It is essential to identify and manage any cause of secondary osteoporosis. Failure to do so may result in further bone loss despite pharmacologic intervention. A few exams, together with the history of the patient, can exclude up to 90 % of secondary osteoporosis (table III in supplemental material) [18].

**Table:**
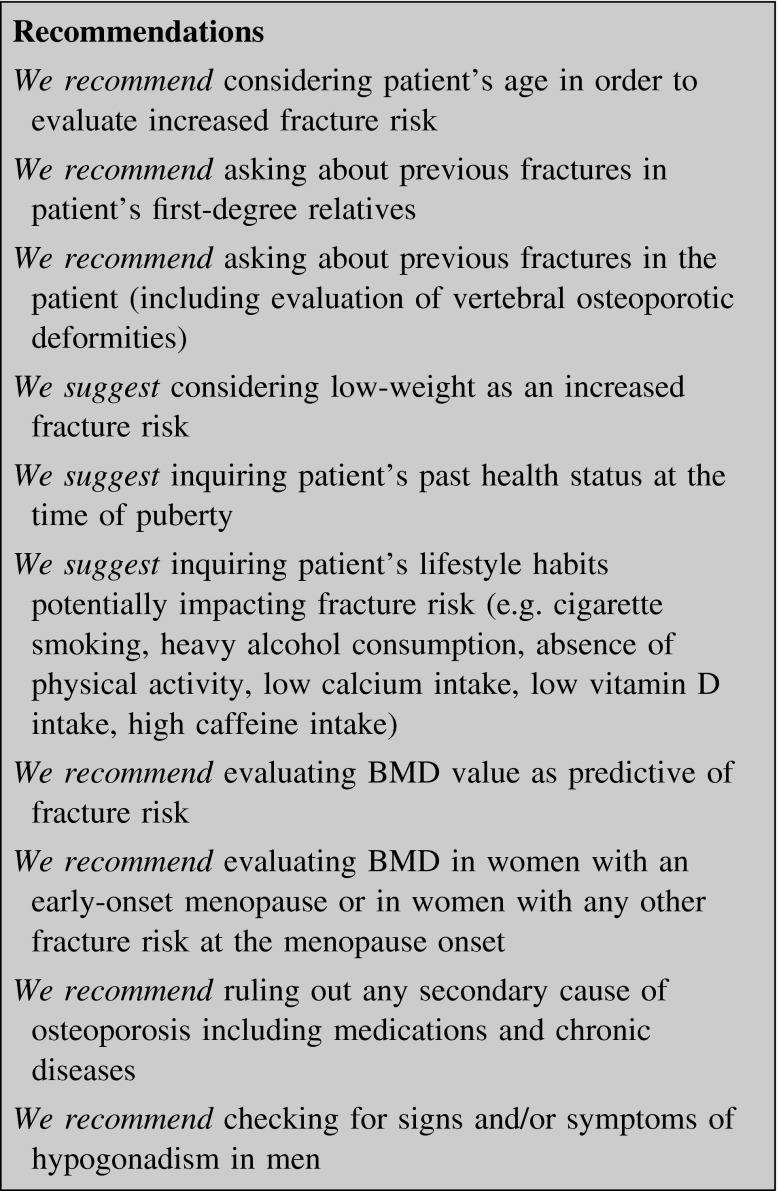

#### Treatment thresholds and indications to treatment

Even though fracture risk is higher in osteoporotic women, the great bulk of fractures occur in the far largest population of osteopenic women [19]. Many osteoporotic patients with a prior fracture are not tested with a dual-energy X-ray absorptiometry (DEXA) exam, even if these high-risk patients are most likely to benefit from osteoporosis treatment.

FRAX^®^, an algorithm approved by the WHO for estimating the 10-year risk probability of hip and other major osteoporotic fracture [20], should be the tool to identify those patients candidate to pharmacological intervention [21]. FRAX has been validated in different countries (Italy is validating DeFRA, a FRAX derived algorithm) but there is no universally accepted fracture risk level for osteoporosis pharmacological therapy. Therefore, intervention thresholds vary from country to country [22, 23], depending on treatment cost-effectiveness, reimbursement issues, and health care system [24, 25].

Guidelines from National Osteoporosis Foundation and American Association of Clinical Endocrinologists (AACE) continue to recommend treatment of patients with hip and vertebral fractures and those with a central DEXA T-score ≤−2.5, but since 2008 they also began to recommend treatment of patients with DEXA-based T-scores between −2.5 and −1 if they had 10-year hip fracture probability ≥3 % or major osteoporotic-related fracture probability ≥20 % [26, 27].

In any clinical situation fracture risk should be assessed only if its evidence would influence the treatment [28].

**Table:**
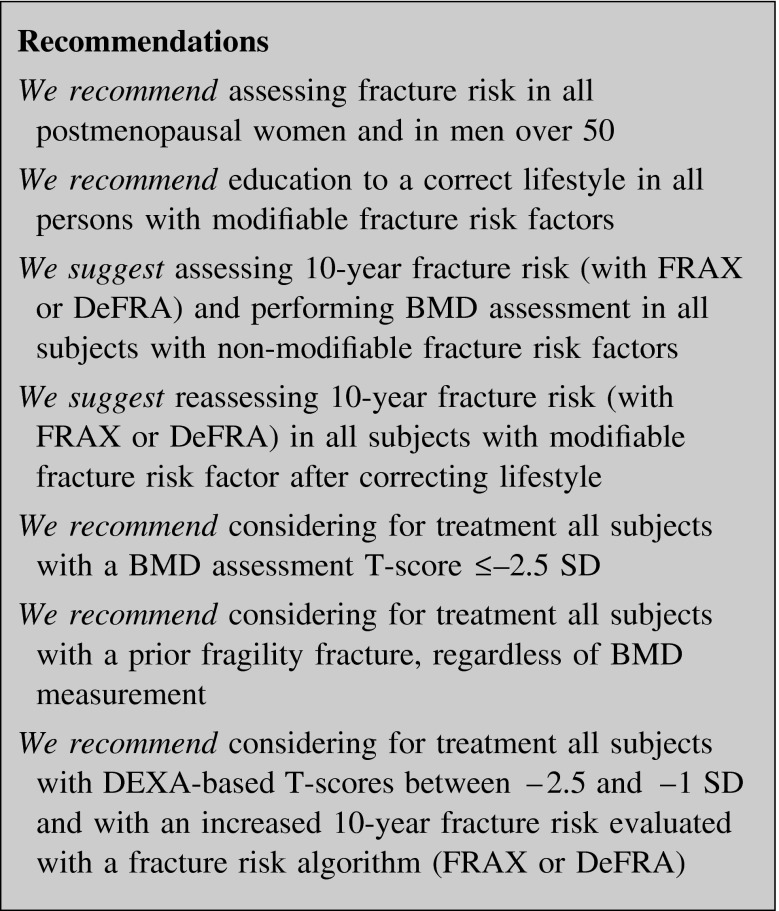

### How to treat

Since all the registration trials, demonstrating the effectiveness of therapies for osteoporosis, associated calcium and vitamin D to the active treatment, it is mandatory to include calcium and vitamin D in every pharmacological treatment strategy.

#### Available drugs

Table 1 lists the drugs commonly used in the treatment of osteoporosis. Etidronate and calcitonin are still on the market, but are not included due to their scarce use.

**Table 1:** Approved drugs for osteoporosis

Class	Molecule	Oral	Injectable
Bisphosphonates	Alendronate	×	
	Risedronate	×	
	Ibandronate	×	×
	Zoledronate		×
	Clodronate	×	×
Strontium ranelate		×	
Anti-RANKL antibody	Denosumab		×
SERMs	Raloxifene	×	
	Bazedoxifene	×	
	Lasofoxifene	×	
Hormone therapy	Estrogens (±progestins)	×	
PTH analogs	Teriparatide		×

##### Bisphosphonates (BPs)

BPs attach to hydroxyapatite binding sites on bone surfaces where they are taken up by osteoclasts during bone resorption. Inside osteoclasts BPs inhibit the farnesyl-pyrophosphate synthase, and impair their ability to form a ruffled border, to adhere to the bone surface and produce protons, thus inducing osteoclasts apoptosis and inhibition of bone resorption [29, 30].

All oral BPs may induce gastrointestinal adverse events, including dyspepsia, dysphagia, and esophageal ulcers [31]. Among tablet formulations it is hypothesized that branded compounds are better tolerate than generics, likely due to differences in the technical production process or in the formulation of excipients [32].

The safety of long-term BPs therapy is unclear. Post-marketing reports of rare but serious adverse events, likely associated with prolonged therapy, such as atypical femoral fractures (AFF), osteonecrosis of the jaws (ONJ) and esophageal cancer, prompted the Food and Drug Administration to perform a systematic review of long-term efficacy and safety of these drugs. Data were conflicting and the committee did not support a regulatory restriction on the duration of drug use [33, 34].

*Alendronate* Alendronate increases BMD and reduces the risk of vertebral and non-vertebral fractures at 3–4 years in women with PMO defined as either having existing vertebral fracture or FN T-score ≤−2.5 [35, 36].

The risk reduction rate is highly significant (*p* < 0.001) for radiologic vertebral fractures (48 %), multiple radiologic vertebral fractures (87 %), any clinical fracture (30 %), and any non-vertebral clinical fracture (27 %). Risk of hip fracture is reduced by 53 % (*p* < 0.005), clinical vertebral fracture by 45 % (*p* < 0.003), and wrist fracture by 30 % (*p* < 0.038). The reduction in risk is apparent since the first year of treatment for clinical vertebral fractures and for any clinical fracture, by month 18 for hip fracture, by month 24 for non-vertebral fracture and by month 30 for wrist fracture [36].

There are few available data for assessing the efficacy of long-term BP use. The evidence regarding continuing treatment beyond 5 years [37] demonstrates that from 5 to 10-year treatment with alendronate leads to a continuous increase in vertebral BMD, stabilization of femoral BMD, and a reduction of incident clinical (symptomatic) vertebral fractures (RR 0.45; 95 % CI 0.24–0.85). No evidence exists on the overall reduction in non-vertebral fractures, due to the limited size of the extension trial and the small numbers of fractures [33, 37].

Data from the FLEX study suggest that patients with low FN BMD (T-score <−2.5) after 3–5 years of treatment with alendronate and those with an existing vertebral fracture show the highest risk for future vertebral fractures, while patients with a FN T-score >−2.0 or without vertebral fracture have a lower risk degree [33, 37].

Alendronate has been licensed in Europe as oral formulation (tablets of 10 mg/day and 70 mg/week) and, more recently, as a drinkable solution and effervescent formulation [31].

*Risedronate* In women with PMO 3 years of 5 mg/day risedronate significantly reduced the risk of vertebral fracture (−41 to 49 %) in two placebo-controlled RCTs [38, 39] since the first year of therapy. It remained reduced through 7 years of treatment [40, 41], although studies did not include a placebo group after 5 years.

Two studies evaluated the effect of risedronate therapy on the risk of non-vertebral fracture: an RCT reported a 39 % reduction [42], while the 33 % reduction reported in the other study was not statistically significant versus placebo [39].

In an RCT enrolling 5445 postmenopausal women (age range 70–79 years), risedronate (5 mg/day) reduced the hip fracture risk by 40 % in women with a densitometric diagnosis of osteoporosis, and by 60 % in the subgroup with prior vertebral fractures, though the latter result derives from a post hoc analysis [43].

A 2-year RCT on early postmenopausal non-osteoporotic women (mean age, 51–52 years) showed that risedronate (5 mg/day) led to a significant BMD increase at lumbar spine (LS, +5.7 %) and femur (+5.4 %) [42].

In older postmenopausal women, lumbar and femoral BMD increased significantly (+4.3 and +2.8 %, respectively) after 3 years of risedronate therapy (5 mg/day) as compared with placebo-treated matched controls [38].

Risedronate therapy for 7 years increased BMD by 11.5 % from baseline [41].

Risedronate has been approved in Europe for the treatment of PMO in oral tablets (5 mg/day, 35 mg/weekly and 75 mg on 2 consecutive days once a month).

*Ibandronate* In a 3-year placebo-controlled study on postmenopausal women (mean age 69 years) with low spinal BMD and vertebral fractures, oral ibandronate (2.5 mg/day) reduced significantly the risk of morphometric vertebral fracture by 52 % but did not reduce the non-vertebral fracture risk in the overall study population. A post hoc analysis showed that the treatment might reduce (−69 %) the risk of non-vertebral fracture in the subgroup of patients with baseline FN T-scores <−3 [44].

Ibandronate significantly increased spinal and femoral BMD (+5.2 and +4.1 %, respectively) in postmenopausal women with low spinal BMD [44].

In early postmenopausal women (mean age 57.6–58.8 years) without osteoporosis a two-year treatment with oral ibandronate (2.5 mg/day) led to a small, though significant, LS and hip BMD increase (+1.9 and +1.2 %, respectively) [45].

No data are available on the effects of long-term ibandronate therapy.

Ibandronate is approved for the treatment of PMO both as a 150-mg tablet once a month and as a 3-mg intra-venous (IV) formulation every 3 months (not reimbursed in Italy).

*Zoledronate* Zoledronate is the most powerful BP: 60 % is taken up by the skeleton after a 15-min IV infusion [46]. Once-yearly IV infusion of zoledronate (5 mg), during a 3-year period, significantly reduced the risk of vertebral, hip and non-vertebral fractures in women with PMO; moreover zoledronate increased BMD at all skeletal sites [47].

The infusion within 90 days after surgical repair of an osteoporotic hip fracture is associated with a reduction of new clinical fractures and improved global survival at 36 months [48].

Many patients can discontinue zoledronate after 3 years, but those at high risk of fracture may benefit by continuing treatment up to 6 years [49].

Flu-like symptoms may occur, particularly during the first administration. Paracetamol or ibuprofen alleviates post-dose symptoms that are commonly transient (lasting 1–3 days). In case of severity and persistence steroids may be helpful [50]. Gastrointestinal problems, local reaction at the injection site and uveitis are minor complications of zoledronate treatment.

Concerns on cardiac action [47] were ruled out by clinical data that did not disclose significant difference in incidence of atrial fibrillation [51].

IV zoledronate is not recommended in subjects with severe renal impairment (creatinine clearance <30 mL/min), but clinical trial showed that IV BPs did not result in long-term renal function decline [52].

ONJ is related to both the potency and duration of BP therapy. Occurrence of ONJ is rare in patients with osteoporosis who are receiving once-yearly zoledronate. Data from five clinical trials indicated that ONJ incidence is <1 in 14,200 treated patient per year [53].

The risk of AFF may rise with increasing duration of exposure to BPs (alendronate or risedronate) while in zoledronate-treated patients the risk is unknown [54].

Zoledronate 5 mg IV yearly is approved for the treatment of PMO.

*Clodronate* A double-blind placebo-controlled trial proved the efficacy of 800 mg daily oral clodronate both in increasing BMD and in reducing the incidence of single and multiple vertebral fractures in a group of women with PMO or secondary osteoporosis, with or without prior vertebral fractures [55]. Oral clodronate is also effective and comparable to other BPs in reducing all clinical fractures in elderly women, while it seems to be less effective on hip fractures [56].

A recent meta-analysis underlined the efficacy of clodronate in reducing the risk of new vertebral, non-vertebral and overall fractures but the majority of the data are from studies with oral clodronate [57].

Oral clodronate given at a dose of 1600 mg/day, for at least 3 years, in women with normal BMD or with osteopenia affected by early stage breast cancer (BC) and treated with adjuvant chemotherapy or anti-estrogen treatment, increased BMD, with effects lasting for 7 years after treatment discontinuation [58].

Weekly 100 mg intramuscular clodronate is registered in Italy for PMO treatment but it is not reimbursed. There are few low-quality studies concerning the effects of this formulation on fractures [59, 60].

Clodronate (generally at an oral daily dose of 1600 mg) is approved in patients with BC and it has been used also in subjects with myeloma and less frequently in other solid tumors with or without bone metastases [61].

Clodronate cannot be a real therapeutic option in Italy because oral formulation has no indication for PMO and at present it is not reimbursed by the Italian National Institute of Health.

**Table:**
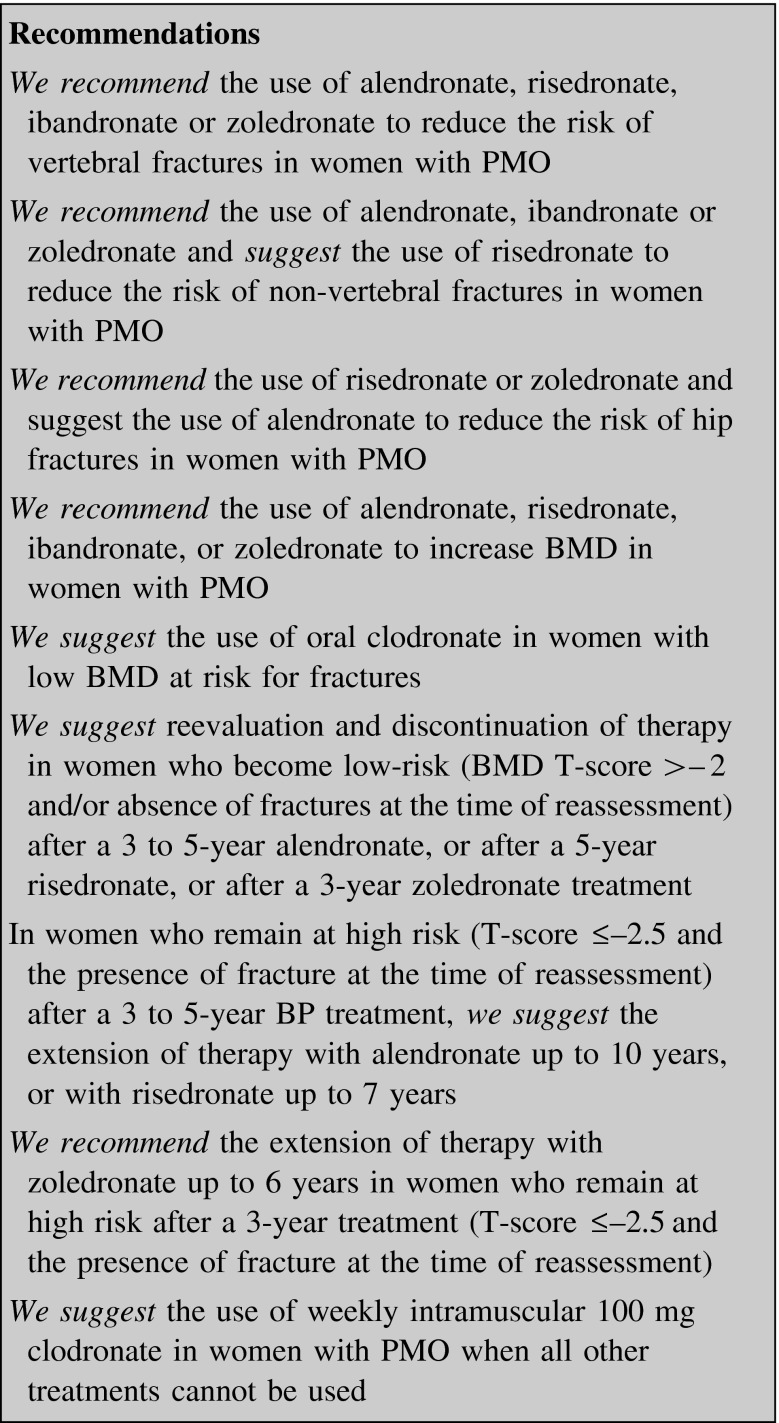

##### Strontium ranelate (SrR)

The mechanism of action of SrR is still not completely known, even though it seems to increase bone formation and reduce bone resorption [62–64].

SrR increases BMD and reduces the risk of vertebral and non-vertebral fractures at 3 and 5 years in women with PMO [62, 63]. It reduces the relative risk of hip fractures in subjects older than 74 years and T-score <−3 [62]. Open-label extension of these trials (up to 10 years) showed further increase in BMD [64].

SrR has been approved in Europe, as an oral 2-g daily formulation, for the prevention of vertebral and non-vertebral osteoporotic fractures in osteoporotic women.

SrR has been associated with an increased risk of venous thromboembolism and with rare cases of severe allergic skin reactions [65]. More recently, long-term post-approval surveillance safety analyses showed an increased cardiovascular risk in patients treated with SrR [66], though not confirmed by other analysis [67, 68]. SrR is now contraindicated in patients with a history of cardiovascular and cerebrovascular disease, and uncontrolled hypertension [69]. Patients should be evaluated for cardiovascular risk before starting treatment with SrR and at regular intervals during treatment [65, 69].

**Table:**
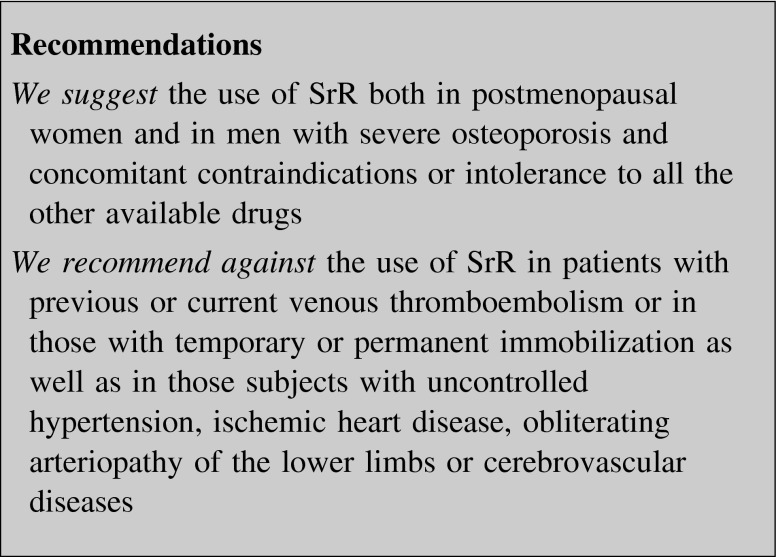

##### Denosumab

RANK ligand (RANKL) induces osteoclastogenesis by binding to its receptor (RANK). The interaction between RANKL and RANK can be blocked by a decoy receptor called osteoprotegerin, as well as by denosumab, a fully human antibody against RANKL. The effect of denosumab is the reduction of formation, function and survival of osteoclasts, which determines both a reduction of bone resorption and an increase of BMD. The drug is administered SC every 6 months. It is not cleared by kidneys, thus it can be used in patients with renal failure [70].

In PMO 36-month denosumab treatment reduced the incidence of vertebral, hip and non-vertebral fractures (−68, −40, and −20 %, respectively) [71]. Denosumab reduced the risk of vertebral, non-vertebral and hip fractures in patients older than 75 years [72, 73].

In the 5-year extension study denosumab induced gains in BMD at LS and total hip (TH) (+13.7 and +7 %, respectively) [74]. In naïve-to-treatment PMO BMD increased more with denosumab than with alendronate [75], and in patients previously treated with BP the switch to denosumab induced a greater increase in BMD [76].

Denosumab is safe and well tolerated. Dermatological adverse events (i.e. dermatitis, rash, eczema, and in particular cellulitis and erysipelas) were more prevalent in treated patients than in placebo, although the number of events was very low (0.3 %). Hypocalcemia has been reported but not in patients assuming calcium and vitamin D. Few cases of ONJ and two of AFF have been reported, while there is no apparent increase of cancer or cardiovascular diseases.

**Table:**
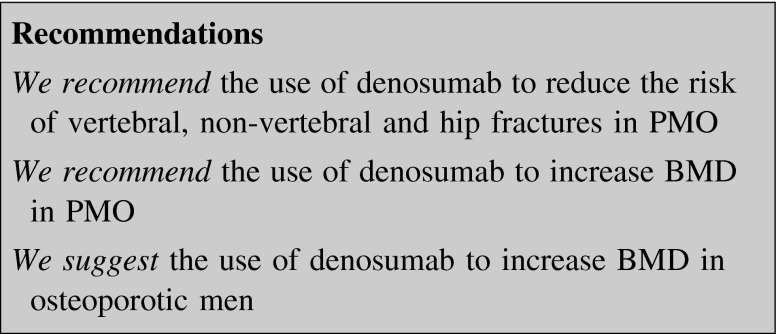

##### Selective estrogen receptor modulators (SERMs)

SERMs are non-steroidal compounds that bind to the estrogen receptor, acting as agonist or antagonist, depending on the target tissue.

Treatment with SERMs is associated with an increased frequency of hot flushes, leg cramps and venous thromboembolic events [77–80]. No adverse endometrial effect is observed with raloxifene and bazedoxifene [81], whereas an increased, but not clinically significant, endometrial thickness is associated to lasofoxifene [82].

*Raloxifene* (60 mg daily) is approved in the USA and Europe for the prevention and treatment of PMO. In a 3-year, placebo-controlled RCT in postmenopausal women with osteoporosis (T-score <−2.5) or low bone mass and prior vertebral fracture, raloxifene decreased the risk of new vertebral fractures by 50 and 30 %, respectively [77]. Raloxifene had no effect on the rate of non-vertebral and hip fracture [77]. Raloxifene showed effectiveness also in increasing BMD and reducing the risk of vertebral fractures in postmenopausal women with osteopenia [83].

*Bazedoxifene* (20 mg daily) is approved in Europe for the treatment of PMO in women at increased risk of fracture. In a 3-year placebo-controlled RCT in PMO, with or without prior vertebral fractures, bazedoxifene decreased the risk of new vertebral fractures by 42 % [84]. In a 2-year extension of the study, bazedoxifene decreased the risk of new vertebral fractures by 35 % [85]. Bazedoxifene had no effect on the rate of non-vertebral fracture and hip fracture. In a 2-year placebo-controlled RCT bazedoxifene associated with conjugated estrogen significantly increased BDM at the LS and hip [86].

*Lasofoxifene* (0.5 mg daily) is approved in Europe for the treatment of PMO in women at increased risk of fracture. In a 5-year placebo-controlled RCT in PMO, lasofoxifene decreased the risk of new vertebral fractures and non-vertebral fractures by 42 and 24 %, respectively, but not hip fractures [77].

**Table:**
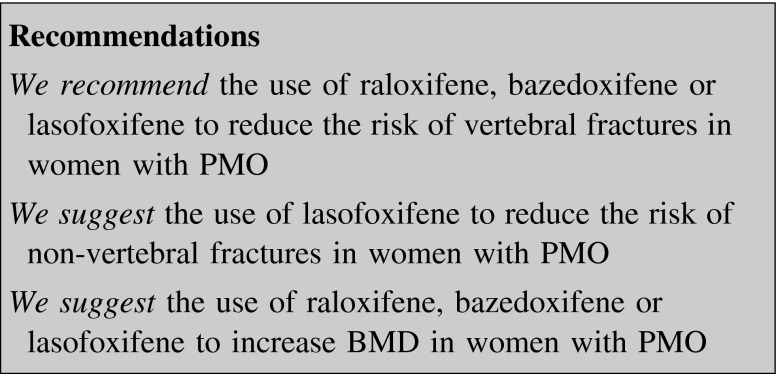

##### Hormone therapy (HT)

Estrogens reduce the accelerated bone turnover induced by menopause and prevent bone loss at all skeletal sites regardless of age and duration of therapy. The beneficial effects of systemic oral or transdermal estrogen therapy (ET) or estrogen plus progestin (EPT) for women with or without a uterus, respectively, on BMD preservation are established. RCTs indicated that standard doses of ET/EPT reduce spine and non-vertebral (including hip) fractures [87–89]. In the Million Women Study, when the overall fracture risk reduction was examined by type of hormone, no difference was found between ET and EPT [90]. Results were not influenced by sequential or continuous progestin use [90]. The RR of fracture was not different when specific estrogen or progestin products were compared (i.e. conjugated estrogens vs. estradiol; medroxyprogesterone acetate (MPA) vs. norethisterone or norgestrel/levonorgestrel) [90].

The benefits of HT on bone mass and fracture reduction dissipate quickly after discontinuation [91, 92], requiring the transition to a different treatment to preserve bone mass. In the Women’s Health Initiative (WHI), women in the EPT/ET group who stopped HT for a few years had a rate of fractures equivalent to that of women assigned to placebo [91, 92]. The long-term risks of HT outweighed the benefits because systemic estrogen and MPA at standard doses for 5.6 years significantly increased risk of BC, stroke, coronary heart disease (CHD) and thromboembolic events [93–95]. In women who had undergone a hysterectomy, ET alone for 6.8 years resulted in a statistically significant increased risk of stroke and deep venous thrombosis, whereas BC, CHD, and pulmonary embolism were unchanged, suggesting a deleterious effect of MPA [96].

**Table:**
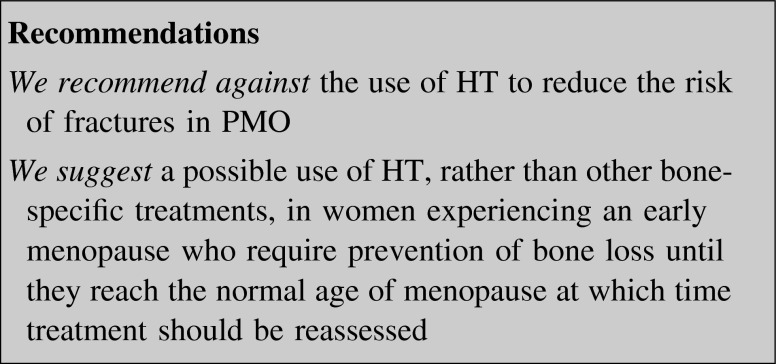

##### Teriparatide

The 1–34 N-terminal fragment of PTH (teriparatide, 20 μg daily by SC injection) is the only approved anabolic therapy for the management of PMO at increased risk of fracture [97]. The duration of treatment is limited to a maximum of 2 years.

Teriparatide is well tolerated, but nausea, pain in limbs, headache and dizziness were reported [98]. Contraindications to treatment include severe renal impairment (GFR <30 mL/min), primary hyperparathyroidism, Paget’s disease, unexplained elevation of alkaline phosphates, prior radiation therapy, skeletal malignancy, and bone metastases. The increased risk of osteosarcoma in rats was not confirmed for the relatively short-term use in humans [99].

In an RCT in women with prevalent vertebral fractures, teriparatide, compared with placebo, decreased the risk of new vertebral and non-vertebral fractures by 65 and 35 %, respectively, after a median 19-month treatment [100]. The beneficial effect on fracture risk persisted after discontinuation of therapy for 18 months for vertebral fractures and 30 months for non-vertebral fractures (both 40 % reduction vs. placebo) [101, 102].

BP administration upon termination of teriparatide may strengthen the beneficial effect of therapy [101–103]. The efficacy of teriparatide may be variably attenuated by prior treatment with anti-resorptive drugs according to their potency [104–109].

**Table:**
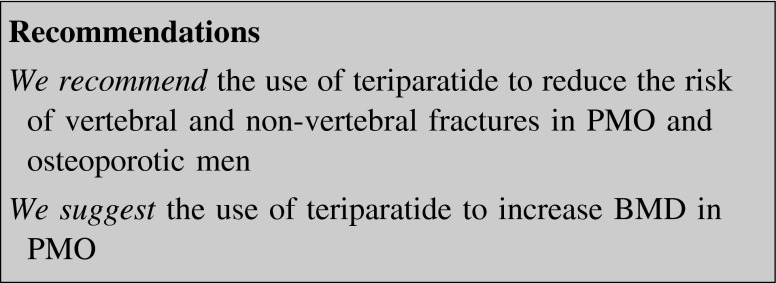

Table 2 summarizes the efficacy of different drugs on fracture risk in PMO.

**Table 2:** Efficacy of different treatments on fracture risk in postmenopausal women (from 36, 38, 39, 42, 43, 44, 47, 48, 55, 62, 63, 71, 72, 73, 77, 79, 84, 100)

Drug	Vertebral	Non-vertebral	Hip
Alendronate	+	+	+
Risedronate	+	+	+
Ibandronate	+	±	–
Zoledronate	+	+	+
Clodronate (800 mg/day, orally)	+	+	–
Strontium ranelate	+	+	±
Denosumab	+	+	+
Raloxifene	+	–	–
Bazedoxifene	+	±	–
Lasofoxifene	+	+	–
Teriparatide	+	+	–

Beware that results are not to be regarded as comparative between different drugs, since they are not derived from head to head studies

− no available data or negative data, ± fracture risk decrease reported only in post hoc analyses, + fracture risk decreased vs. placebo

#### Monitoring the effectiveness of treatment

##### Bone turnover markers (BTMs)

BTMs may show a large and rapid response to the pharmacological treatment of osteoporosis. BTMs may offer an attractive monitoring strategy as they are non-invasive, relatively cheap, and able to detect changes in bone metabolism earlier than and independent from BMD variations [110]. As all studies have limitations due to significant pre-analytical and analytical variability of BMTs, the International Osteoporosis Foundation and the International Federation of Clinical Chemistry and Laboratory Medicine recommended the standardization of one marker of bone formation (if possible N-terminal propeptide of procollagen type 1, P1NP) and one of bone resorption (if possible serum collagen type I C-telopeptide, CTX) [110].

Almost all the published studies focused on the effectiveness of BTMs in predicting BMD changes during osteoporosis therapy [111], but the strength of this association was poor [112, 113].

Scanty data correlated fracture risk reduction with variation of BTMs; weak evidence supports their role in monitoring anti-resorptive therapy [62, 114–117], as well as in measuring fracture risk reduction during treatment with teriparatide or SrR [62, 117]. Nevertheless bone resorption markers seem to be more accurate than formation ones in predicting both bone loss rate and fracture risk reduction [118].

Evidence is still lacking about the threshold at which BTMs should be considered over-suppressed and potentially related with complications such as AFF.

**Table:**
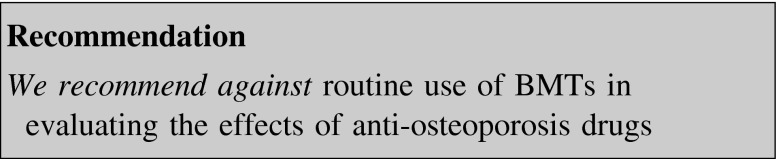

##### Bone mineral density

BMD testing of central skeletal sites (spine and hip) by DEXA is considered the international standard for detecting osteoporosis, determining the risk of fracture, identifying the candidates to treatment and monitoring the efficacy of drugs. The goal of drug therapy for osteoporosis is to significantly increase bone strength, with BMD being one of the major determinants of this parameter [119], as well as to reduce fractures.

Changes in BMD reportedly account only for a small part of the fracture risk reduction seen in patients treated with anti-resorptive drugs [120]. In the FIT Study, spine BMD increase could explain only 16 % of the risk reduction of vertebral fracture [112]. In patients treated with risedronate or raloxifene the changes in BMD correlated even more poorly with the degree of vertebral or non-vertebral fracture risk reduction. As a matter of fact patients taking risedronate, regardless whether their LS or hip BMD was increased or not, showed a similar incidence of non-vertebral fractures [121]. Similarly, raloxifene-induced BMD increase was able to explain only 4 % of the observed vertebral fracture risk reduction, with the remaining 96 % being unexplained [122]. A higher correlation was described for teriparatide, whose action on BMD could explain from 30 to 41 % of vertebral fracture risk reduction [123].

More clear data were published on denosumab and SrR, suggesting that a larger proportion of their anti-fracture efficacy might be explained by changes in TH or FN BMD. A risk reduction up to 35 % for new or worsening vertebral fractures and up to 87 % for non-vertebral fractures can be explained by denosumab-induced increase of BMD [124]. SrR changes in hip and FN BMD have been correlated with a vertebral fracture risk reduction by 74 and 76 %, respectively [125].

BMD monitoring may help in identifying non-responder or non-compliant subjects; DEXA should be measured 1–2 years after starting or changing any anti-osteoporosis therapy [126]. These intervals are based both on the observed rates of change induced by anti-resorptive agents and on the reproducibility of DEXA testing. Once efficacy has been established, less frequent intervals between DEXA scans seem appropriate.

Whether greater treatment-related changes in BMD result in greater decreases in fracture risk is controversial [127], but BMD testing alone seems to predictably underestimate the extent of fracture risk reduction induced by therapy. Other determinants of bone strength but BMD, like bone geometry, microarchitecture, remodeling rate, damage accumulation and collagen/mineral matrix properties, play an important role in reducing fracture risk.

**Table:**
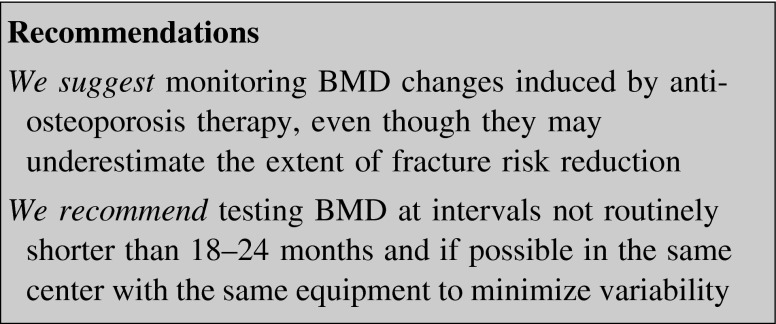

### Conducting the treatment

#### Defining a non-responder

A number of targets were proposed in osteoporosis treatment and this makes possible to assess a treatment failure [128, 129]. BTMs and changes in BMD are the main candidates for these targets. Another controversial point is that, since drug therapy does not eliminate fracture risk, sustaining a single fracture during treatment should not be considered failure, as the risk of a new fracture decreases significantly during the treatment [130, 131].

Regarding BTMs, in a post hoc analysis of the FIT study, osteoporotic women with higher baseline levels of P1NP, CTX and bone-specific alkaline phosphatase (BSAP) showed a greater reduction in risk of non-vertebral (but not vertebral) fractures in response to alendronate than those with low levels [132]. Major reductions in one or more BTMs were reportedly associated with substantial reductions in vertebral, non-vertebral and hip fractures in women treated with alendronate [114]: the greater the reduction in BTMs, the lower is the risk of fracture. FIT study showed that women in the alendronate group, with a reduction of at least 30 % of BSAP, had a lower risk of non-vertebral and hip fractures. This effect was just as strong as the anti-fracture effect observed with changes in BMD after 1 year [114]. A <25 % fall in serum CTX (the least significant change, LSC, at 95 % confidence) on an anti-resorptive drug or teriparatide should be considered a treatment failure [110].

Much more controversy exists in relation to target BMD values during osteoporosis treatment. Some studies suggested that even patients with a decline in BMD might still benefit from oral BP therapy [133]. In clinical trials of alendronate, risedronate, and ibandronate, 8–25 % of patients had BMD change ≤0 % at the LS after 2 years of treatment. Post hoc analyses suggested that patients who experienced an increase in BMD had a lower vertebral fracture risk than those with a decline in BMD (range 38–50 %). Additional analyses suggested that patients who experienced a decline in BMD while receiving oral BP therapy still appeared to receive some benefit (fracture risk reduction, 38–60 %) from the treatment compared with those on a placebo [133]. It may, therefore, be difficult to tell a patient on BP treatment with a declining BMD over time that he/she has really lost his/her protection against osteoporotic fractures. Thus, a decline in BMD ≥5 % at the LS and ≥4 % at the FN (corresponding to LSC at 95 % confidence) or a new fragility fracture should be considered treatment failure, as well as a reduction in serum CTX on anti-resorptive drugs or an increase on teriparatide smaller than LSC for the used marker.

**Table:**
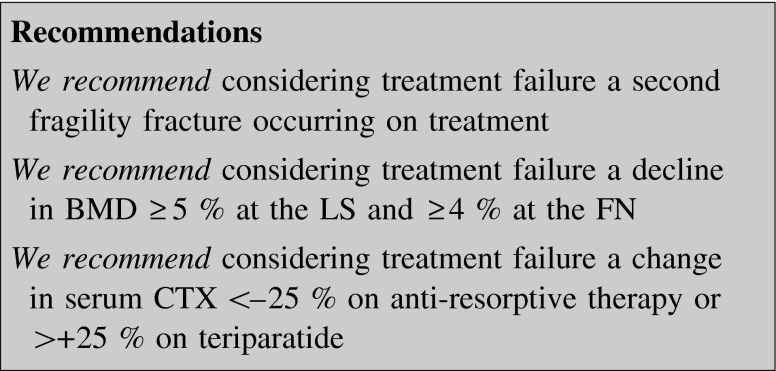

#### How long to treat

BPs continue to be the first-line agents to treat osteoporosis due to their efficacy, low cost, possible extra-skeletal effects [134], and the possibility of a residual effect following cessation of therapy [37, 49]. On the other hand, BP-associated AFF, although rare, may cause a considerable impact on the patient’s quality of life and should also be considered in planning when to stop or change therapy [54, 135–137]. This is mainly due to the fact that after 5 years of BP treatment, there is a sharp increase from 0.13 to 0.22 % in the risk of AFF [136].

There are extension studies on alendronate, zoledronate and risedronate showing that patients may experience a residual effect after stopping therapy (“drug holiday”) [37, 41, 49]. The benefit of continuing alendronate beyond 5 years has been observed for clinical vertebral fracture only [37]. Taken together, the rates of osteoporotic fractures were similar in patients who continued or stopped alendronate after a 5-year treatment [138]. On the other hand, a post hoc analysis demonstrated that patients with both no prevalent vertebral fracture at the start of FLEX trial and a BMD T-score ≤−2.5 would derive much more benefit in continuing therapy as regards clinical vertebral fracture risk reduction (number needed to treat in 5 years: 24) [33]. Likewise, women with a prevalent vertebral fracture at the start of FLEX trial and a BMD T-score ≤−2.0 derived a greater benefit when remaining on alendronate [33]. Women who were on 5 mg/day during FLEX study had a similar benefit to those on a 10-mg dose, suggesting that alendronate doses of less than 70 mg per week might be considered for treatment beyond 5 years [33].

The anti-fracture efficacy of risedronate was maintained for up to 7 years of treatment, and women who switched from placebo to risedronate at 5 years significantly decreased the incidence of vertebral fractures during the sixth and seventh years [41]. An extension study with zoledronate beyond 3 years showed that women who continued annual infusions for up to 6 years had a 49 % reduction in the risk of morphometric vertebral fractures compared with those who stopped at 3 years [49]. Women with a FN T-score at 3 years ≤−2.5 benefited much more from continuing treatment for 6 years [49].

**Table:**
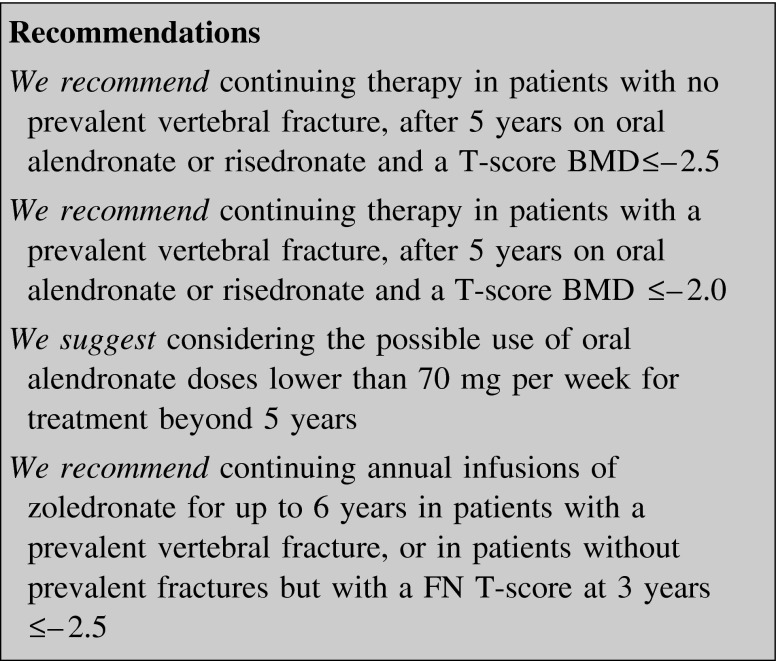

#### Drug switch

In the high-risk patient one alternative is to change therapy to a class of osteoporosis medication with no pure anti-resorptive effect or an osteoanabolic one, that is, SrR or teriparatide, respectively [139–142].

Postmenopausal women switched to SrR after long-term BP use showed different responses in BTMs in comparison to women who never used them [139–141]. There were short-term increases in serum CTX and osteocalcin [139] associated with long-term increases in BMD [140]. During the first 6 months of SrR therapy, there was a blunting of BMD response in patients previously treated with BPs compared to those who were not previously exposed to BPs. However, in the long term, they found a catch-up of BMD in those patients, along with an increase in BTMs (CTX, P1NP, and BSAP) [141].

The anti-fracture efficacy of teriparatide was compared in patients previously exposed to long-term BPs with those who had never used BPs. They were treated with teriparatide for 18 months and followed up for 36 months. All fractures decreased in both groups and this was maintained in the observation period, in which 70 % of patients were on BPs. Back pain and quality of life improved in both groups, but these improvements were more pronounced in those patients with no prior BP use [142].

Since teriparatide administration is limited to 2 years and BMD starts to decrease shortly after its discontinuation, sequential therapy with anti-resorptive agents after teriparatide treatment is highly recommended although the benefit in terms of fracture risk reduction is speculative [27]. Alendronate [143] and, even more, zoledronate [144] and denosumab [145] after teriparatide are associated with a further increase in BMD. Also raloxifene maintains spine BMD and increases hip BMD after teriparatide discontinuation [146].

Not all the patients treated with BPs can be switched to teriparatide due to the high cost of this therapy. A double-blind, double-dummy RCT investigated the effects of switching to denosumab 504 postmenopausal women who had been receiving alendronate therapy for at least 6 months. Transition to denosumab produced greater increases in BMD at all measured skeletal sites and a greater reduction in bone turnover than did continued alendronate therapy [76].

**Table:**
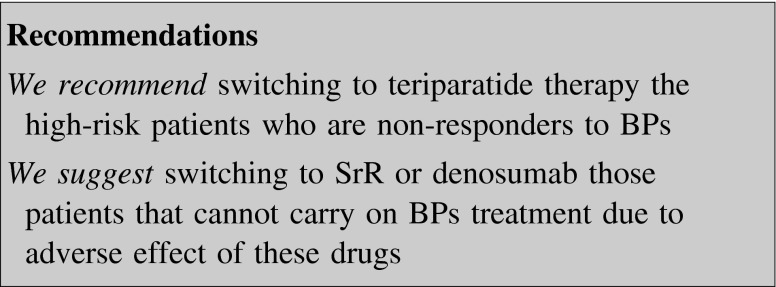

#### Adherence

In a cross-sectional, observational study in 2314 women with PMO, the rates of noncompliance to drug treatment were: alendronate 14.9 %, risedronate 11.4 %, raloxifene 11.3 %, HT 24.1 %, and tibolone 16.7 % [147]. The rates of gastrointestinal side effects leading to noncompliance were similar for alendronate and risedronate (62.7 vs. 62.2 %) [147].

Retrospective data showed that compliance with osteoporosis guidelines by physicians is not adequate [148]: among women diagnosed with PMO, only 13.2 % had appropriate BMD follow-up, 42 % were not on approved and guideline-endorsed pharmacotherapy for osteoporosis, and 26 % had a fracture after diagnosis.

As adherence seems to be a problem in osteoporosis treatment both by the patient and the physician, the great variability in methods of studies that evaluate the impact of compliance to drug therapy on fracture risk makes it somewhat difficult to draw any definite conclusions on this issue [149].

**Table:**
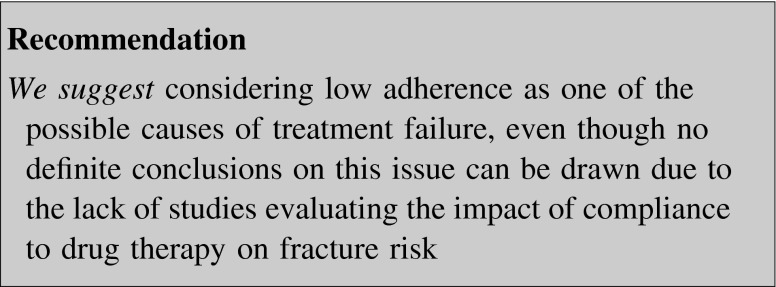

## Other forms of osteoporosis

The present document focuses on forms representing a challenge for the endocrinologist. Glucocorticoid-induced osteoporosis (GIO) has not been addressed because many recent documents appeared on the topic.

### Male osteoporosis

The incidence of vertebral and hip fractures is lower in men than in women. Anyway, it has been estimated that after 50 years one out of four men will suffer a fragility fracture [150]. Morbidity and mortality after hip fractures are higher in men than in women, particularly after the age of 70 [151].

Since male osteoporosis has a secondary etiology in many cases, before starting a drug treatment it is mandatory to differentiate the various forms of osteoporosis by means of an accurate clinical evaluation of the patient (table II in supplemental material) [11].

Currently the indications for drug therapy are not unequivocal. The use of algorithms that integrate risk factors with BMD may be useful in assessing fracture risk [20]. Pharmacological treatment must be carefully considered in men with a prior history of hip or vertebral fractures, particularly if they occurred after minor traumas or after the age of 50, or in those on long-term glucocorticoid therapy or having a FN BMD T-score ≤−2.5 [152].

At present many pharmacological treatments can be considered for osteoporosis in men: T, BPs (alendronate, risedronate, zoledronate), denosumab, and teriparatide.

#### Testosterone

T is the first-line treatment in men affected by primary or secondary hypogonadism [153]. Although the prevalence of hypogonadism in osteoporotic men has not been properly assessed, it is reasonable to measure serum T if concomitant symptoms or signs of androgen deficiency are present or suspected, subsequently considering for androgen treatment only patients with T levels <200–300 ng/dL [154].

In men over 65 years T therapy was able to increase spine BMD by 3.4 % when a threshold of 300 ng/dL was considered, while this rate grew up to 5.9 % when plasma T threshold was set at 200 ng/dL [155]. No data are available on anti-fracture efficacy of T [156].

#### Bisphosphonates

A good quality trial has proven the efficacy of a 2-year daily oral alendronate therapy in increasing BMD. Although the study was not powered to assess anti-fracture efficacy, a not significant trend to reduction in the number of vertebral and non-vertebral fractures was observed in the treated group [157].

Similar data on BMD have been obtained with weekly oral risedronate, but even with this drug fracture incidence was not significantly different between risedronate and placebo-treated patients [158, 159].

In a 2-year multicenter, double blind RCT, once-yearly zoledronate (5 mg IV) increased BMD and decreased BTMs, comparably to 70 mg weekly oral alendronate, with similar incidence of adverse events [160, 161].

More recently in a multicenter, double-blind, placebo-controlled RCT zoledronate treatment was associated with a significantly reduced risk of vertebral fracture among men with primary or hypogonadism-associated osteoporosis. The treated group showed also higher BMD and lower BTMs [162].

The effects of BPs on BMD seem similar in men with normal serum T and in those with hypogonadism, but a recent paper on HIV-infected men, with osteoporosis or osteopenia, showed that risedronate increased BMD and reduced BTMs to a greater extent in patients with adequate androgenization compared to patients with symptomatic hypogonadism [163].

#### Denosumab

Denosumab is effective in reducing fracture incidence in men receiving ADT for non-metastatic prostate cancer (see below). Denosumab 60 mg SC every 6 months resulted in BMD increase significantly higher than placebo (+5.7 % at the LS, +2.4 % at the TH, and +2.1 % at the FN) in a randomized, 1-year study in osteopenic men with or without fractures [164].

A 24-month extension of this study, the ADAMO trial, evaluated the efficacy and safety of denosumab for the treatment of men with low BMD. After the first year men from the original denosumab group continued to receive the drug for an additional year, while those coming from the placebo arm were assigned to the treatment with denosumab. This treatment for a second year maintains the ability to increase BMD, together with reductions in bone resorption. In men initiating denosumab during the second year BMD increased as well. The effects on BMD and BTMs were similar to those reported in women with PMO and in men with prostate cancer receiving ADT [165].

#### Teriparatide

Teriparatide is indicated to increase bone mass in men with primary or hypogonadal osteoporosis at high risk for fracture. A placebo-controlled, double-blind RCT with teriparatide was conducted on 437 men with low BMD and a 40 % prevalence of fractures. Almost 50 % of the enrolled patients had low serum T. BMD increase was significantly higher at all skeletal sites in the teriparatide arm of the study. Changes in BMD were similar to those described in women with PMO. Bone turnover increased significantly, with formation markers rising earlier than resorption ones. Treatment effects were independent on the presence of baseline low serum T [166].

**Table:**
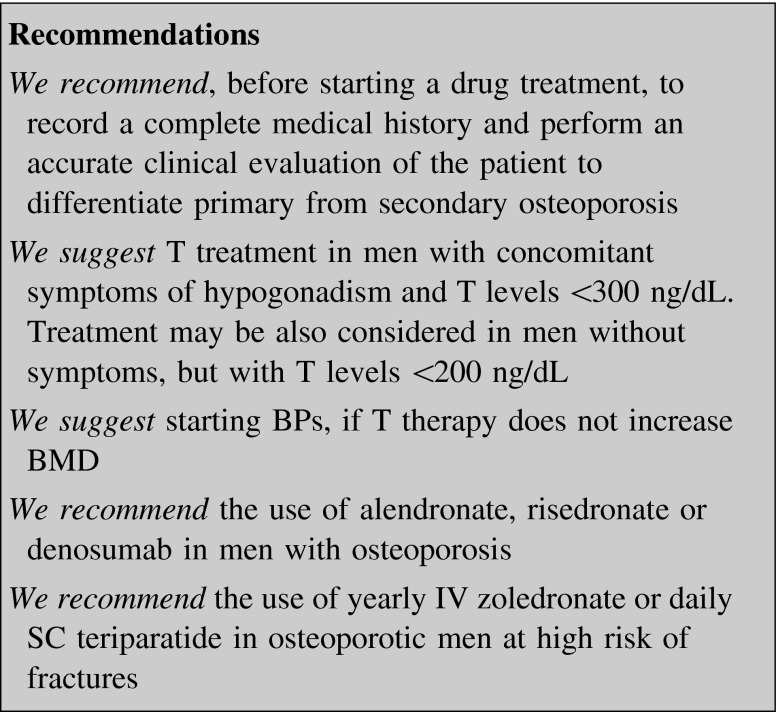

Table 3 summarizes the efficacy of different drugs on fracture risk in males.

**Table 3:** Efficacy of different treatments on BMD and fracture risk in males (from 156, 157, 158, 159, 160, 162, 165, 167)

Drug	BMD increase	Fractures		
		Vertebral	Non-vertebral	Hip
Testosterone	Yes	–	–	–
Alendronate	Yes	±	–	–
Risedronate	Yes	±	–	–
Zoledronate	Yes	+	–	–
Denosumab	Yes	±	–	–
Teriparatide	Yes	±	–	–

Beware that results are not to be regarded as comparative between different drugs, since they are not derived from head to head studies

− no available data or negative data, ± fracture risk decrease obtained as secondary endpoint, + fracture risk decreased vs. placebo

### Androgen deprivation therapy

ADT in men induces a severe sex-steroid deficiency resulting both in decreased BMD (at the hip and spine levels) and increased fracture incidence [167–173]. Radius BMD decline is faster than that at the spine and hip [174].

Men receiving ADT should receive drug therapy if they have a high risk of fracture as follows [151]:

- Low trauma hip or vertebral fractures;
- BMD T-score of the spine, FN and/or TH ≤−2.5 SD;
- Long-term glucocorticoid therapy at pharmacological doses.

Other studies are needed to establish the correct therapeutic approach for men receiving ADT who have a T-score between −1.0 and −2.5 at the spine, FN, or TH level, using national fracture risk assessment algorithms.

Physical exercise, calcium, vitamin D, BPs, denosumab, and SERMs have been proposed as treatment options for men on ADT.

#### Physical exercise

There are no conclusive data about the real effect of physical exercise on BMD and risk of fracture in men on ADT. A recent systematic review demonstrated that physical exercise may ameliorate many of the treatment-induced adverse effects of ADT, but its impact on bone health is still unclear [175]. Another cross-sectional study revealed a weak positive and significant association between endurance exercise and T-score of the hip. No significant correlation was found between physical exercise and LS T-score, even after adjusting for age, BMI and alcohol use [176].

#### Calcium/vitamin D

In men receiving ADT, calcium and vitamin D supplementation is controversial with inconsistent dosage recommendations. A critical review, analyzing the results of 12 clinical trials of calcium and vitamin D supplementation on BMD in men on ADT, showed the ineffectiveness of the commonly recommended doses, namely 0.5–1 g daily calcium plus 200–500 IU daily vitamin D [177]. In a prospective longitudinal study, vitamin D, but not calcium, seemed to increase LS BMD during the first year of ADT [178]. On the other hand, reduced daily calcium intake potentially represents an independent risk factor for osteoporosis in these patients [179].

#### Bisphosphonates

Treatment with BPs is able to prevent bone loss in men on ADT. However, most studies evaluated the ability of different BPs in preventing BMD decline, but they were not powered to evaluate fracture risk reduction [180–182].

Zoledronate was able to increase BMD in men with or without bone metastases treated with ADT [183, 184].

In a phase 3 double-blind placebo-controlled RCT on men treated with ADT, oral once-weekly 70 mg alendronate increased both LS and TH BMD [185].

Oral risedronate significantly recovered BMD for up to 24 months compared with the control group after starting ADT [186].

In an open-label study, IV pamidronate prevented hip and LS bone loss in men treated with ADT [187].

Finally, also neridronate prevented ADT-induced bone loss in a small RCT in patients with prostate cancer [188].

#### Denosumab

Denosumab has been approved for prevention of treatment-related fragility fractures in men on ADT. In a double-blind multicenter placebo-controlled trial, denosumab (60 mg q 6 months) determined a significant reduction of new vertebral fractures incidence at 36 months and an increase of BMD at all skeletal sites [174].

#### SERMs

SERMs are effective in ameliorating BMD and reducing fracture risk in men on ADT, but they are not approved for this use probably due to a poor risk/benefit ratio. Raloxifene treatment increased hip BMD in a 12-month open-label study in men on ADT [189]. In a phase 3 double-blind placebo-controlled trial, toremifene both improved BMD at all skeletal sites and reduced the incidence of new morphometric and clinical vertebral fractures, but it also increased the rate of venous thromboembolic events [190].

**Table:**
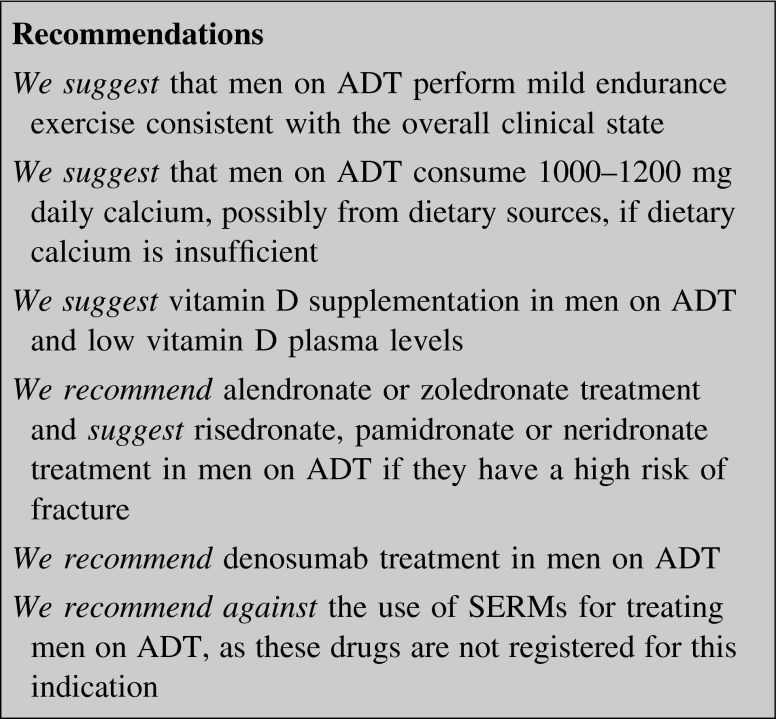

### Premenopausal osteoporosis

Osteoporosis in premenopausal women is rare and it is usually due to other conditions or diseases or is of genetic origin (i.e. osteogenesis imperfecta, Marfan syndrome, Gaucher’s disease) [191–193]. The pathophysiology of idiopathic osteoporosis is not well understood. Pregnancy-associated osteoporosis is a rare idiopathic form of spinal osteoporosis or transient osteoporosis of the hip. Preexisting low BMD and high bone turnover rate both during pregnancy and lactation may play an important pathophysiologic role [194].

Medical history, physical examination and laboratory tests should be performed to detect secondary causes of osteoporosis (table II in supplemental material). History of low trauma fractures is diagnostic for osteoporosis, but it is extremely rare in premenopausal females. The diagnosis of osteoporosis can be made even without fracture history, if a low BMD and a secondary cause of osteoporosis are both present.

The 2007 International Society for Clinical Densitometry Official Position recommends the use of BMD Z-score instead of T-score in premenopausal women. For *Z*-score values <−2.0 a definition of “BMD below the expected range for age” should be used [195]. However, low BMD alone does not represent a pathological state [196] and it is not sufficient to diagnose osteoporosis since relationships between BMD and fracture incidence have not been established in premenopausal women [197–199].

Finally, it should be reminded that fracture prediction tools such as the FRAX^®^ are not applicable in subjects younger than 40 years.

In case of secondary osteoporosis the first therapeutic approach should be addressed toward the removal or the management of the underlying cause (i.e. parathyroidectomy for primary hyperparathyroidism, gluten free diet for celiac disease, nutritional rehabilitation and weight gain for nervous anorexia).

When a “BMD below the expected range for age” is diagnosed, behavioral advices are mandatory. Weight-bearing exercise, adequate intake of calcium and vitamin D, smoking cessation and avoidance of excess alcohol are suggested, albeit no evidence is available on their real effect in reducing fracture rate [200].

BPs are the most studied drugs and they may be prescribed in the treatment of premenopausal osteoporosis, particularly in presence of fragility fractures. Anti-fracture efficacy of BPs has not been sufficiently documented in this setting, except for neridronate, pamidronate and risedronate in osteogenesis imperfecta [201–203]. More positive results have been obtained when BMD increase was considered as an endpoint of BP treatment in some conditions (i.e. inflammatory bowel diseases, nervous anorexia, cystic fibrosis, beta-thalassemia, osteogenesis imperfecta, Gaucher’s disease), even though these studies have both small size and short duration (usually <24 months) [201–209].

The most frequent form of secondary osteoporosis that might require treatment in premenopausal women is GIO. Patients taking 7.5 mg of daily glucocorticoids for more than 3 months with a prior history of fracture should be treated with BPs. Teriparatide should be used in patients with a higher risk of fracture or in those whose response to treatment is considered inadequate [210].

BPs administration during pregnancy could potentially lead to congenital malformations, [211–213]. Since BPs accumulate and remain for years in the skeleton, precaution is advised for their use before pregnancy and during lactation [214, 215].

**Table:**
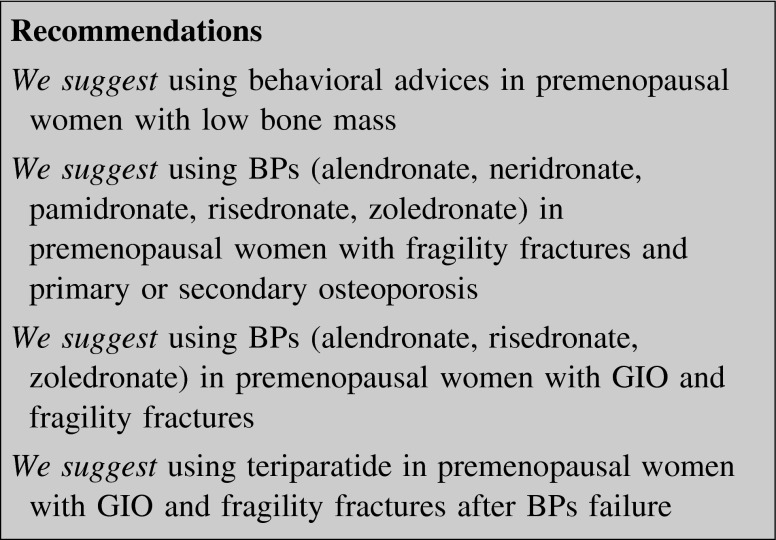

### Aromatase inhibitors

Premature ovarian suppression induced by GnRH agonists, chemotherapy, surgical removal, or irradiation is the first cause of bone damage in premenopausal women with BC, with an estimated bone loss of about 8 % within the first year [216]. In addition, both in pre- and postmenopausal women, AI can decrease BMD and increase fragility fracture incidence compared to tamoxifen [217–220]. In a study comparing anastrozole with tamoxifen the overall incidence of fractures was higher in the former group (11.0 vs. 7.7 %; OR 1.49; 95 % CI 1.25–1.77) [221].

The extent of bone loss is different for the various molecules at LS or TH, reaching a 5-year reduction for anastrozole of −6.1 and −7.2 %, respectively [218]. Letrozole and exemestane can induce a 2-year reduction in BMD by −5.3 and −2.4 % at LS, respectively, whereas the respective figures were −3.6 and −1.8 % at TH [222, 223]. Over 30 % of the patients starting on anastrozole will have a diagnosis of osteoporosis in the subsequent years [218].

Several RCTs indicate that both oral and IV BPs as well as denosumab have protecting effects on BMD in this setting. Zoledronate has shown a higher efficacy than oral BPs. In premenopausal women on anastrozole plus goserelin therapy the concomitant use of zoledronate (4 mg IV every 6 months) was able to improve BMD at 5 years, compared to the group treated only with tamoxifen plus goserelin [217].

The three “Zometa-Femara Adjuvant Synergy Trials” have compared the efficacy of zoledronate in postmenopausal women receiving adjuvant letrozole. The patients were randomly assigned to immediate zoledronate (4 mg IV q 6 months for 5 years), or zoledronate therapy starting after a decrease in BMD or a non-traumatic fracture. At 60 months patients in the immediate group showed a LS and TH BMD gain of +4.3 and +1.6 %, respectively, while the respective figures in the delayed group were −5.4 and −4.2 %. Also the patients with a normal baseline BMD (T-score >−1) showed a substantial decrease in LS (−7.1 %) if they were enrolled in the delayed group, while the immediate group achieved a 3.9 % increase [224–226]. Similar results were obtained in a fourth study, where patients had previously been treated with tamoxifen for 6 years and were then switched to letrozole [227].

Oral risedronate (35 mg weekly) given to postmenopausal BC women treated with anastrozole increased BMD at 24 months (+2.2 to 5.7 % at LS and +1.6 to 1.8 % at TH) [228, 229]. In osteopenic postmenopausal women given anastrozole, patients treated with risedronate gained +1.1 % BMD at LS compared with a 2.6 % decrease in those without BP [230].

In patients treated with anastrozole or letrozole, alendronate significantly increased LS BMD by 3 % after a 6-month treatment [231]. Finally in the 24-month Arimidex–Ibandronate study, monthly 150 mg oral ibandronate prevented bone loss in osteopenic women and in a small number of women with PMO [232].

Although all studies on BPs were not designed for fracture incidence, a recent meta-analysis found that zoledronate (4 mg q 6 months) reduced significantly the overall fracture rate (OR 0.78; 95 % CI 0.63–0.96) [233].

Denosumab, 60 mg q 6 months SC for 24 months in postmenopausal women with low bone mass, was associated with larger BMD gains than placebo across multiple skeletal sites regardless of patient subgroups (prior tamoxifen use, duration and type of AI, time since menopause) [234, 235]. Denosumab reduced the risk of vertebral fractures after 36 months of treatment compared to placebo [236].

On the basis of the AI effects on bone loss and fracture risk, BMD should be measured before AI treatment or within 6 months from treatment start and known risk factors for fracture (table I in supplemental material) should be explored.

Treatment should be initiated in the presence of a fragility fracture irrespectively of BMD values, as well as in case of a baseline T-score <−2.0 SD, or if the annual rate of bone loss results >4 % at LS or TH, or in women combining a T-score <−1.0 SD with more than one of the risk factors for fracture, and finally in women >65 years regardless of BMD [237–240].

In AI-treated patients the adverse event rates were similar to those described in the studies on primary osteoporosis, both for BPs and denosumab. The optimal duration of treatment has not been established but is reasonable to continue anti-resorptives until AI treatment is ongoing.

**Table:**
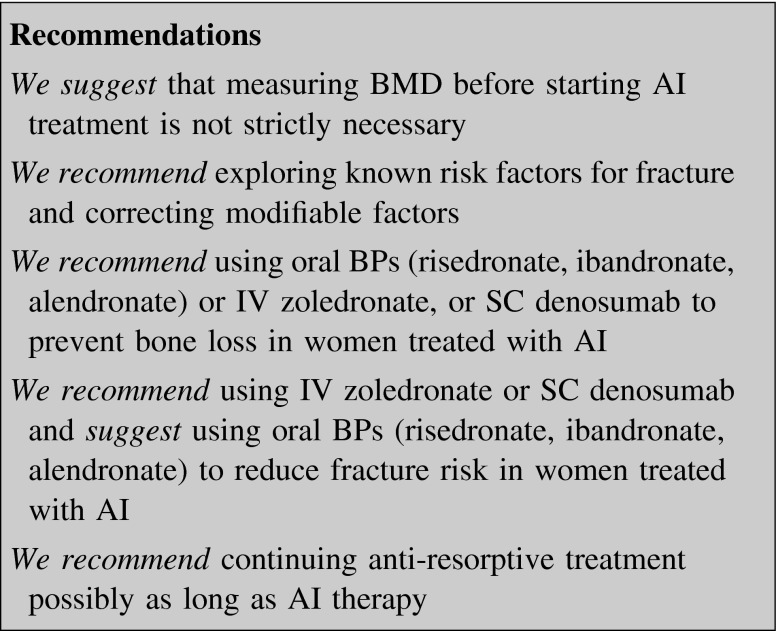

## Conclusions

Removal of modifiable risk factors as well as calcium and vitamin D levels optimization is a pre-requisite for any pharmacological strategy for osteoporosis treatment. An appropriate screening of secondary forms of osteoporosis is mandatory in the appropriate clinical setting. Start of treatments known to be detrimental to bone health, namely glucocorticoids, ADT and AI, should prompt a comprehensive treatment plan to preserve bone health or minimize the damage.

Several drugs are available for the treatment of PMO. Almost all demonstrated a high anti-fracture efficacy together with a good safety profile, leading to a positive risk/benefit balance. Whereas most have proven to significantly reduce the occurrence of vertebral fractures, some discrepancies remain regarding the level of evidence related to their non-vertebral or hip anti-fracture effect.

HT is no longer indicated for the management of PMO, but it might be considered for a limited length of time in the early postmenopausal woman with climacteric symptoms, with a careful individual evaluation of risk–benefit ratio.

SERMs might be a first-line option in early, asymptomatic, postmenopausal women, but could be also considered in women <65 years at high risk of vertebral fractures.

BP administration represents an effective choice in postmenopausal women >65 years at risk of vertebral fractures. Alendronate and risedronate could also be considered in women with established osteoporosis and high risk of hip fractures. Oral administration should be avoided in patients with delayed esophageal emptying and in those who are unable to maintain an upright position. These patients, and those with gastrointestinal adverse events, are candidates for IV zoledronate. Treatment can be continued for up to 5 years; the use for a longer period should be considered with caution. A drug holiday has been suggested because of the persistent anti-fracture efficacy of these drugs after withdrawal, but treatment can be continued in those with very high risk of fracture.

Denosumab might be the first choice in patients with renal failure and high risk of fractures, and after failure or adverse events of other treatments. Hypocalcemia must be corrected prior to denosumab administration, particularly in patients with severely impaired renal function. Patients should be monitored for the risk of infections.

Because of the intermittent administration, zoledronate (once a year) and denosumab (every 6 months) may be an attractive option for patients with low adherence to drugs.

The use of SrR should be restricted to patients with severe osteoporosis, who are intolerant to other treatments, provided that there is no history of prior or concomitant cerebral or cardiovascular diseases.

Treatment with teriparatide should be reserved to established osteoporosis when prior therapies have failed and may be started soon after prior treatment withdrawal. Teriparatide should not be used in patients with hypercalcemia, neoplastic and metabolic bone diseases other than osteoporosis and should not be extended over 24 months. Anti-resorptive therapy (BPs or denosumab) should be started upon termination of teriparatide to maintain its beneficial effects.

In conclusions, most women with PMO may need long-term treatment and the sequential use of different drugs. An individualized approach should be defined when treatment is started taking into account the fracture risk, the efficacy, safety, convenience and life expectancy. Pharmacoeconomic issues and country-specific rules may influence the therapeutic strategy. The treating physician should choose the most suitable treatment for the individual patient based on medical history, fracture risk, drugs anti-fracture efficacy, presence of co-morbidities, previous treatment for osteoporosis, and preference of individual patients. Figure depicts a flowchart for the management of osteoporosis.

## Electronic supplementary material

Below is the link to the electronic supplementary material.

Supplementary material 1 (DOCX 13 kb)

## Abbreviations

AACE: American Association of Clinical Endocrinologists
ADT: Androgen deprivation therapy
AFF: Atypical femoral fractures
AI: Aromatase inhibitors
BC: Breast cancer
BMD: Bone mineral density
BMI: Body mass index
BP: Bisphosphonate
BSAP: Bone-specific alkaline phosphatase
BTM: Bone turnover marker
CHD: Coronary heart disease
CI: Confidence interval
CTX: Collagen type I C-telopeptide
DeFRA: Derived fracture risk assessment tool
DEXA: Dual energy X-ray absorptiometry
ET: Estrogen therapy
EPT: Estrogen plus progestin therapy
FIT: Fracture intervention trial
FLEX: FIT long-term Extension
FN: Femoral neck
FRAX: Fracture risk assessment tool
GFR: Glomerular filtration rate
GIO: Glucocorticoid-induced osteoporosis
GnRH: Gonadotropin-releasing hormone
GRADE: Grading of recommendations, assessment, development, and evaluation
HIV: Human immunodeficiency virus
HT: Hormone therapy
IV: Intravenous
LoE: Level of evidence
LS: Lumbar spine
LSC: Least significant change
MPA: Medroxyprogesterone acetate
ONJ: Osteonecrosis of the jaw
OR: Odds ratio
P1NP: N-terminal propeptide of procollagen type 1
PMO: Postmenopausal osteoporosis
PTH: Parathyroid hormone
RANK: Receptor activator of nuclear factor kappa B
RANK-L: RANK ligand
RCT: Randomized controlled trial
RR: Relative risk
SD: Standard deviation
SERM: Selective estrogen receptor modulator
SrR: Strontium ranelate
SC: Subcutaneous
T: Testosterone
TH: Total hip
WHI: Women’s health initiative
WHO: World Health Organization
