# Supplementary material for: Italian association of clinical endocrinologists (AME) position statement: drug therapy of osteoporosis
Source: J Endocrinol Invest. 2016 Mar 11;39:807–34. doi: 10.1007/s40618-016-0434-8 (PMC4964748; doi:10.1007/s40618-016-0434-8)
Supplement: Supplementary file 1 — Supplementary material 1 (DOCX 13 kb) [file 40618_2016_434_MOESM1_ESM.docx]

**8.** **SUPPLEMENTAL MATERIAL**

| **Table I**  **Fracture risk factors** | |
| --- | --- |
| **Non-modifiable** | Diseases affecting bone at puberty  Ageing  Female gender  Premature menopause (<40 years)  Family history of osteoporosis or fracture in first-degree relatives  Personal history of fracture as an adult |
| **Modifiable** | Low calcium and/or vitamin D intake  Sedentary lifestyle  Cigarette smoking  Heavy alcohol consumption  High caffeine intake  Low BMI (<18 kg/m^2^)  Estrogen deficiency |

| **Table II**  **Secondary causes of bone loss and increased fracture risk** | |
| --- | --- |
| **Medications** | Glucocorticoids (>3 months)  Aromatase inhibitors (AI)  Cytotoxic agents  Anticonvulsants  Excessive thyroxine doses  GnRH agonists or analogs  Heparin  Immunosuppressive agents (cyclosporine)  Antiretroviral therapy |
| **Endocrine diseases** | Cushing’s syndrome  Hypogonadism (primary and secondary)  Hyperthyroidism  Primary hyperparathyroidism  Diabetes mellitus  Acromegaly  Growth hormone deficiency |
| **Genetic disorders** | Hemochromatosis  Hypophosphatasia  Osteogenesis imperfecta  Cystic fibrosis  Thalassemia |
| **Disorders of calcium balance** | Hypercalciuria  Vitamin D deficiency |
| **Gastrointestinal diseases** | Malabsorption syndromes  Chronic liver diseases  Inflammatory bowel disease  Pancreatic insufficiency  Billroth 1 gastroenterostomy  Total gastrectomy |
| **Other disorders** | Rheumatoid arthritis  Systemic lupus erythematosus  Systemic sclerosis  Ankylosing spondylitis  Lymphoma and leukemia  Multiple myeloma  Anorexia nervosa  HIV  Renal tubular acidosis  Chronic renal failure  Chronic obstructive pulmonary disease  Systemic mastocytosis |

| **Table III**  **First line examinations to rule out secondary osteoporosis** |
| --- |
| Erythrocyte sedimentation rate  Blood cells count  Serum protein electrophoresis  Serum calcium  Serum phosphate  Total alkaline phosphatase  Serum creatinine  24h urinary calcium  Serum testosterone (only in men) |
